# Small-Pox and Vaccination at Leicester

**Published:** 1894-09-15

**Authors:** James Grey Glover


### SMALL-POX AND VACCINATION AT LEICESTER.

By JAMES GREY GLOVER, M.D.

[*Reprinted from the "Times."*]

THE lawless state of Leicester *in re* the Vaccination Acts is no doubt a very sad thing in this law-abiding country and in this enlightened age. But, as it exists, it seems almost a pity that the sanitary authorities should not complete the experiment they seem so proud of. Even they shrink from the last test of faith in their own doctrines. They shrink from mere reliance on sanitary police measures. Such measures are in Leicester carried out in a very thoroughgoing manner, whether legally or not. There are other compulsions than those of law; and in Leicester they know how to use them, and have good reason to use them vigilantly. But there is one thing yet lacking in their courageous experiment. The first line of their defence is a cordon of revaccinated persons round every case that occurs in the town. The medical officer is revaccinated; the sanitary inspectors are revaccinated; the nurses are revaccinated, and (tell it not in Gath!) the other persons in the house of the small-pox case are not only compelled (not by law) to keep themselves to themselves, but are revaccinated. I ascertained these facts from the medical officer, and the then ex-medical officer of health a few years ago, when acting as a commissioner of the *Lancet*. They are systematically kept out of sight by the anti-vaccinators of Leicester, with what fairness I shall leave the public to judge. I desire to avoid every inflammable word or charge. My simple object in writing is to propose a challenge to the Leicester authorities. I propose that they dismiss every vaccinated and revaccinated person from attendance on the small-pox cases that arise, and meet every case by officers and nurses that are not protected by vaccination or revaccination. I submit that with the views they express, this challenge should have no terror for them. Gentlemen who, without any expression of regret or self-blame, bear the legal and moral responsibility of the perfectly preventable death of fourteen unvaccinated children from small-pox should not hesitate in the interest of life and truth to carry lawlessness out to its completion. Till they do so they are no more independent of vaccination for immunity from small-pox than any other community. London till a few months ago was almost free from small-pox, and had been for years. But the Marylebone outbreak came to remind us that it was still a terror to be dealt with, and the rush to the neglected vaccination stations was pitiful. I repeat my challenge. If Leicester really wishes to be taken seriously in controversy let her send unvaccinated persons to meet the cases of small-pox.

